# Supplementary material for: Content-rich biological network constructed by mining PubMed abstracts
Source: BMC Bioinformatics. 2004 Oct 8;5:147. doi: 10.1186/1471-2105-5-147 (PMC528731; doi:10.1186/1471-2105-5-147)
Supplement: Additional File 2 — The original results of the above study (non-essential files are deleted to keep the file size under the limit set by BMC bioinformatics). [file 1471-2105-5-147-S2.bz2 › chilibotAdditionalFile2/dip05/196ID8652557E679/html/ERCC3_ERCC2.html]

 


 **ERCC3** and **ERCC2** 
  
Found 122 abstracts in PubMed, retrieved 05.  
 

 What does Google say? 
 PDF only 
| .edu only 

---

**Interactive relationship** (e.g. stimulation, inhibition, etc)

**Inhibitory relationship**- Moreover, when XPD  [ **ERCC2** ]  mutations prevent interaction with the p44 subunit of TFIIH  [ **ERCC3** ] , transactivation directed by certain nuclear receptors is inhibited, regardless of TTD versus XP phenotype, thus explaining the overlapping symptoms.  Ref: 12820975 Mol Cell, 2003
**Neutral relationship**- S. cerevisiae RAD25  [ **ERCC3** ]  SSL2, RAD3, RAD26, YKU80 HDF2 and RAD54 are the homologue for XPB  [ **ERCC3** ]   **ERCC3** , XPD  [ **ERCC2** ]   **ERCC2** , CSB ERCC6, Ku80 XRCC5 and ATR X HX2, respectively.  Ref: 12638184 Kokuritsu Iyakuhin Shokuhin Eisei Kenkyusho Hokoku, 2002
- General transcription factor IIH TFIIH  [ **ERCC3** ]  consists of nine subunits cyclin dependent kinase 7 Cdk7, cyclin H and MAT1 forming the Cdk activating kinase or CAK complex, the two helicases Xpb  [ **ERCC3** ]  Hay and Xpd  [ **ERCC2** ] , and p34, p44, p52 and p62 refs 1 3.  Ref: 12853965 Nature, 2003

**Non-interactive relationship** (e.g. studied together, co-existance, homology, etc.)

- Here we show that the Drosophila TFIIH  [ **ERCC3** ]  component Xpd  [ **ERCC2** ]  negatively regulates the cell cycle function of Cdk7, the CAK activity.  Ref: 12853965 Nature, 2003
- The antibody study indicated that they thus altered the conformation of one side of TFIIH  [ **ERCC3** ] , consisting of p44, XPD  [ **ERCC2** ] , and Cdk activating kinase subunits, that is essential for the transition stage.  Ref: 12665589 Mol Cell Biol, 2003
